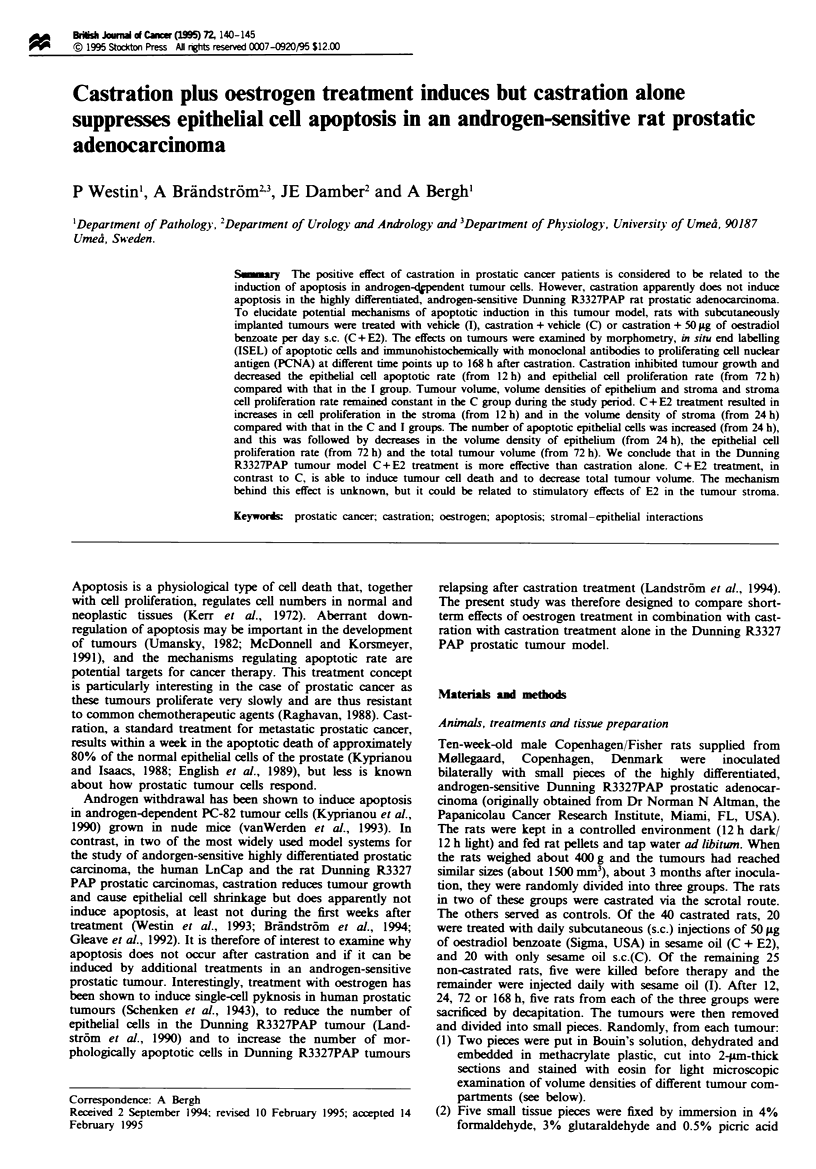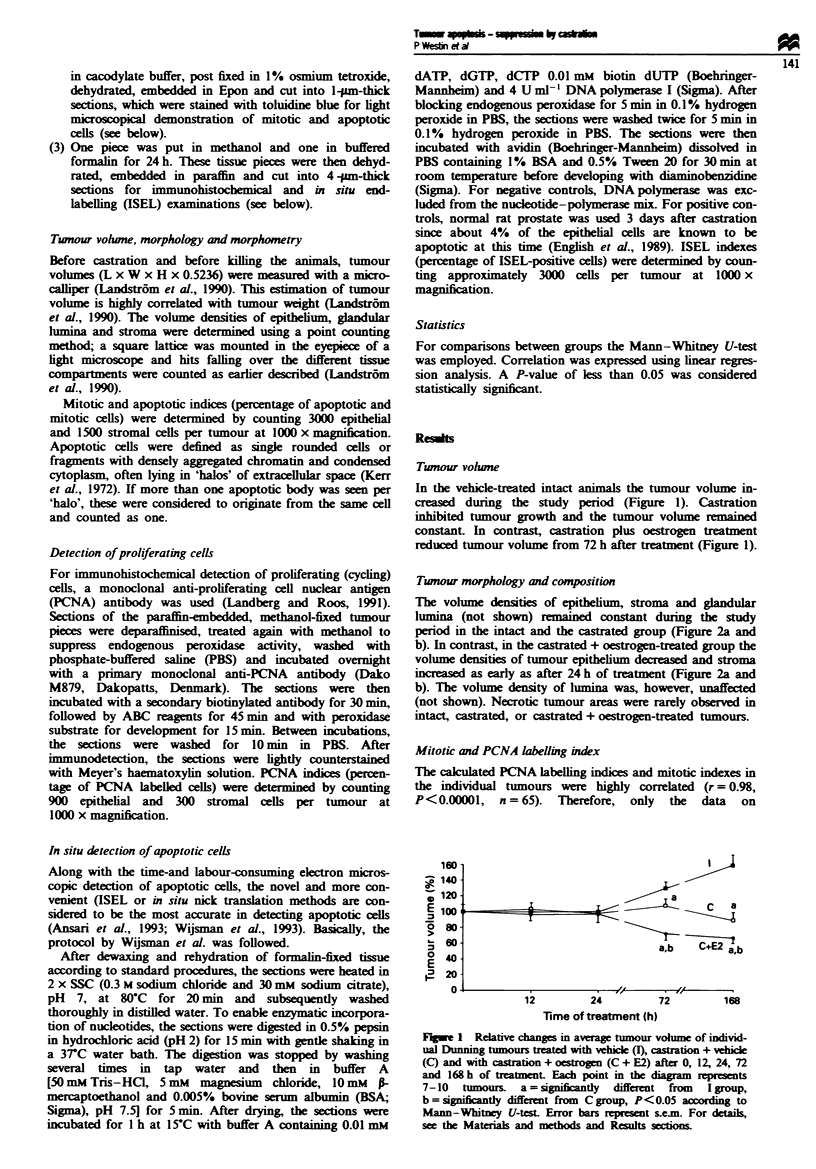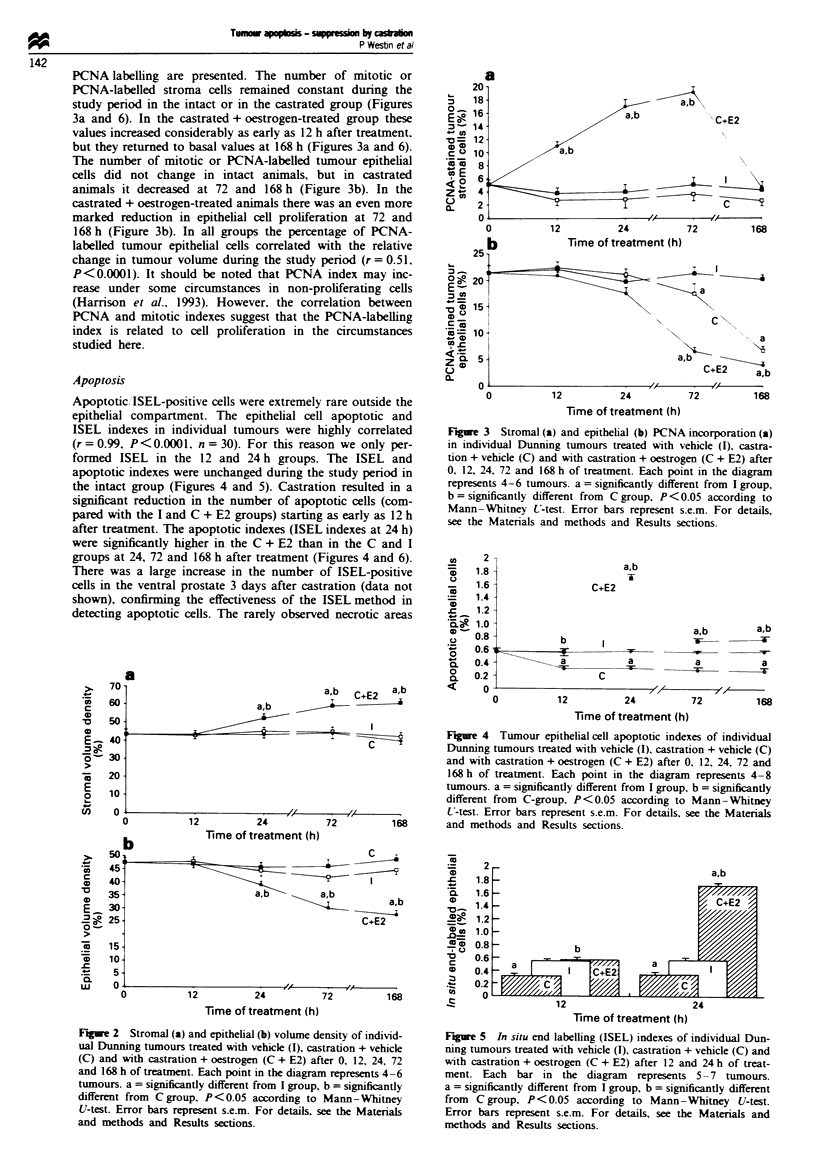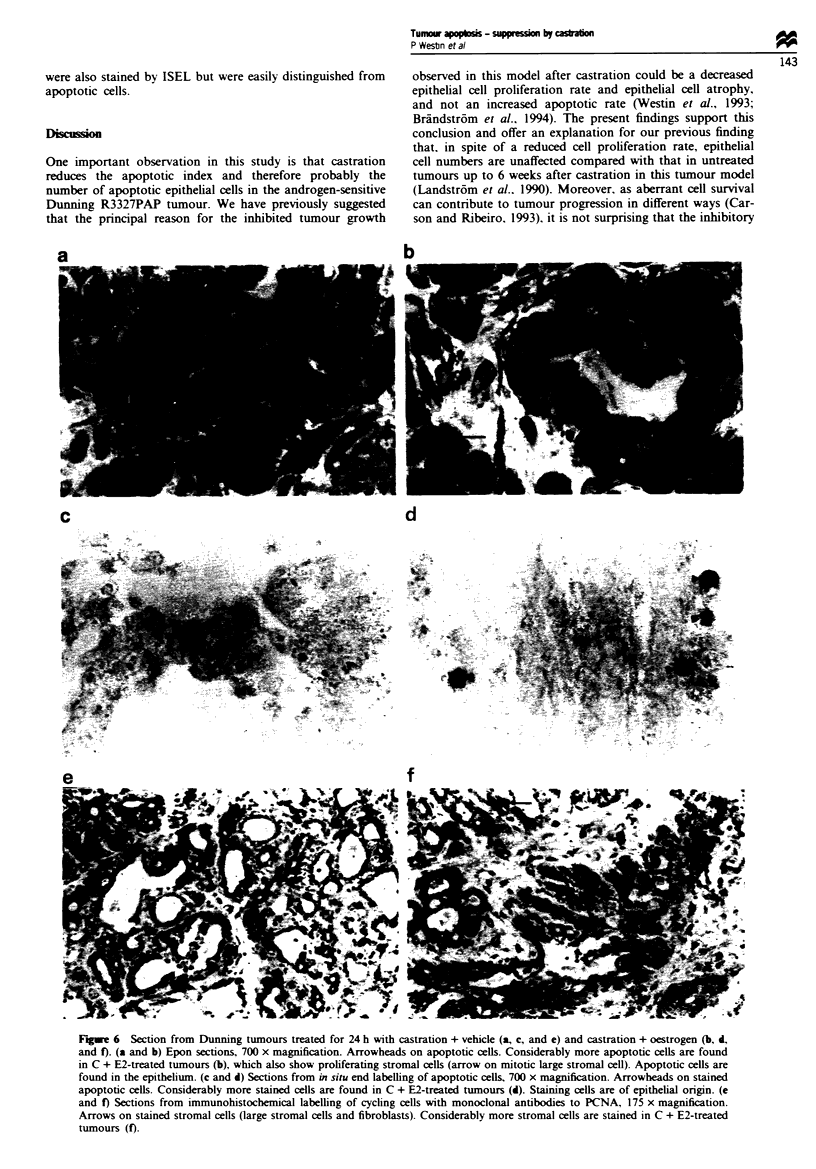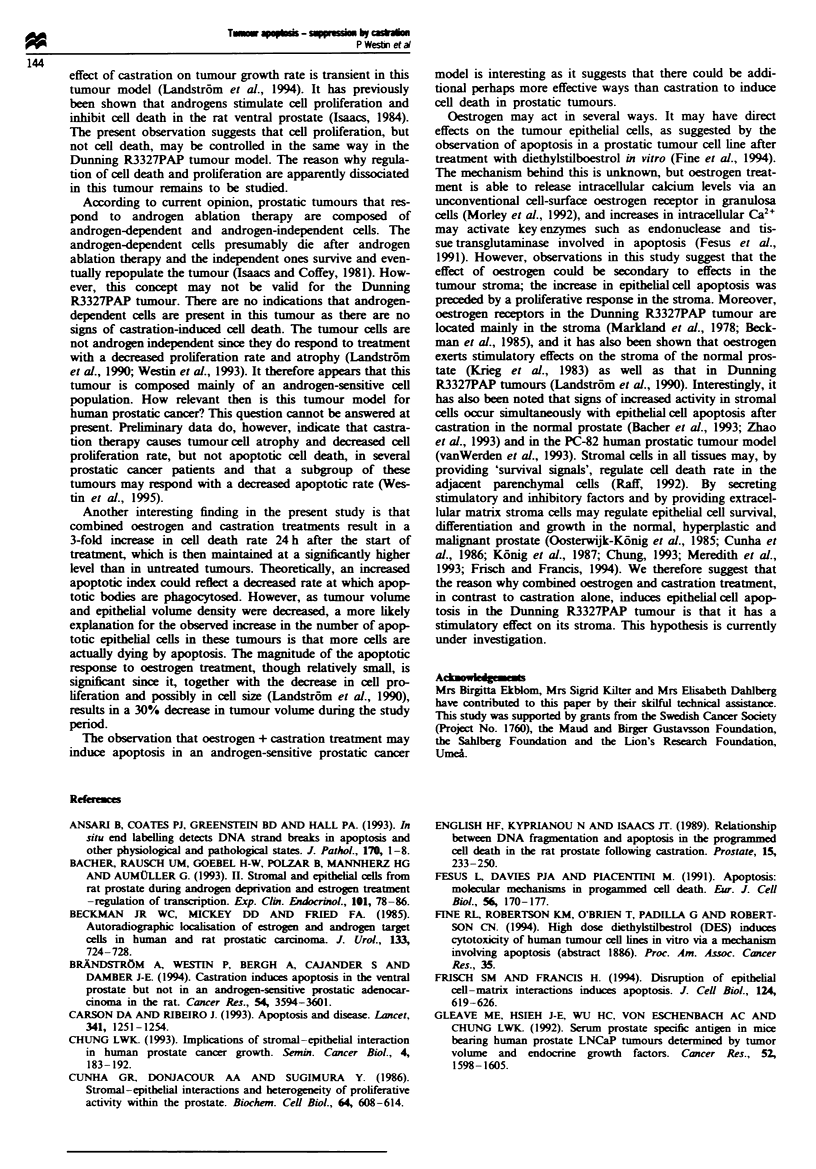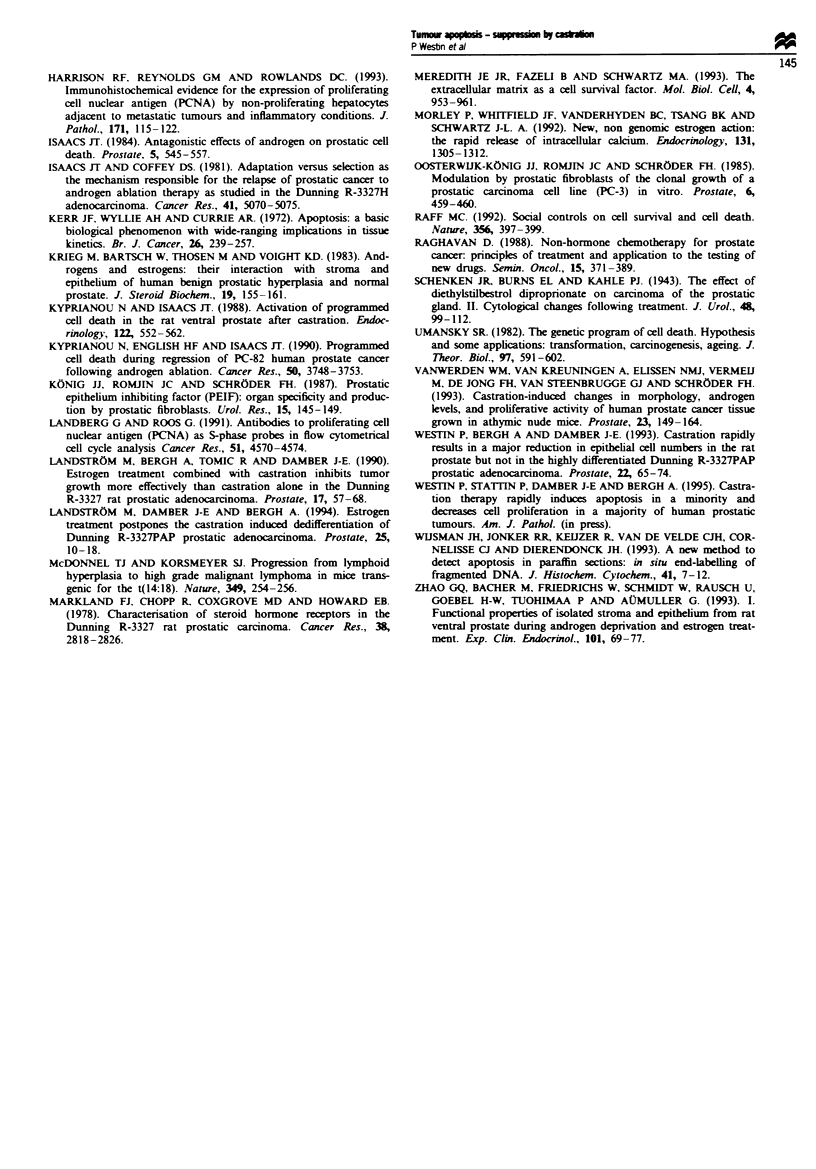# Castration plus oestrogen treatment induces but castration alone suppresses epithelial cell apoptosis in an androgen-sensitive rat prostatic adenocarcinoma.

**DOI:** 10.1038/bjc.1995.290

**Published:** 1995-07

**Authors:** P. Westin, A. Brändström, J. E. Damber, A. Bergh

**Affiliations:** Department of Pathology, University of Umeå, Sweden.

## Abstract

**Images:**


British Journal of Cancer (1995) 72, 140-145

© 1995 Stockton Press All rights reserved 0007-0920/95 $12.00

### Castration plus oestrogen treatment induces but castration alone suppresses epithelial cell apoptosis in an androgen-sensitive rat prostatic adenocarcinoma

P Westin[1], A Brändström[2,3], JE Damber[2] and A Bergh[1]

[1]*Department of Pathology,* [2]*Department of Urology and Andrology and* [3]*Department of Physiology, University of Umeå, 90187 Umeå, Sweden.*

**Summary** The positive effect of castration in prostatic cancer patients is considered to be related to the induction of apoptosis in androgen-dependent tumour cells. However, castration apparently does not induce apoptosis in the highly differentiated, androgen-sensitive Dunning R3327PAP rat prostatic adenocarcinoma. To elucidate potential mechanisms of apoptotic induction in this tumour model, rats with subcutaneously implanted tumours were treated with vehicle (I), castration + vehicle (C) or castration + 50 μg of oestradiol benzoate per day s.c. (C + E2). The effects on tumours were examined by morphometry, *in situ* end labelling (ISEL) of apoptotic cells and immunohistochemically with monoclonal antibodies to proliferating cell nuclear antigen (PCNA) at different time points up to 168 h after castration. Castration inhibited tumour growth and decreased the epithelial cell apoptotic rate (from 12 h) and epithelial cell proliferation rate (from 72 h) compared with that in the I group. Tumour volume, volume densities of epithelium and stroma and stroma cell proliferation rate remained constant in the C group during the study period. C + E2 treatment resulted in increases in cell proliferation in the stroma (from 12 h) and in the volume density of stroma (from 24 h) compared with that in the C and I groups. The number of apoptotic epithelial cells was increased (from 24 h), and this was followed by decreases in the volume density of epithelium (from 24 h), the epithelial cell proliferation rate (from 72 h) and the total tumour volume (from 72 h). We conclude that in the Dunning R3327PAP tumour model C + E2 treatment is more effective than castration alone. C + E2 treatment, in contrast to C, is able to induce tumour cell death and to decrease total tumour volume. The mechanism behind this effect is unknown, but it could be related to stimulatory effects of E2 in the tumour stroma.

**Keywords:** prostatic cancer; castration; oestrogen; apoptosis; stromal-epithelial interactions

Apoptosis is a physiological type of cell death that, together with cell proliferation, regulates cell numbers in normal and neoplastic tissues (Kerr *et al.*, 1972). Aberrant down-regulation of apoptosis may be important in the development of tumours (Umansky, 1982; McDonnell and Korsmeyer, 1991), and the mechanisms regulating apoptotic rate are potential targets for cancer therapy. This treatment concept is particularly interesting in the case of prostatic cancer as these tumours proliferate very slowly and are thus resistant to common chemotherapeutic agents (Raghavan, 1988). Castration, a standard treatment for metastatic prostatic cancer, results within a week in the apoptotic death of approximately 80% of the normal epithelial cells of the prostate (Kyprianou and Isaacs, 1988; English *et al.*, 1989), but less is known about how prostatic tumour cells respond.

Androgen withdrawal has been shown to induce apoptosis in androgen-dependent PC-82 tumour cells (Kyprianou *et al.*, 1990) grown in nude mice (vanWerden *et al.*, 1993). In contrast, in two of the most widely used model systems for the study of andorgen-sensitive highly differentiated prostatic carcinoma, the human LnCap and the rat Dunning R3327 PAP prostatic carcinomas, castration reduces tumour growth and cause epithelial cell shrinkage but does apparently not induce apoptosis, at least not during the first weeks after treatment (Westin *et al.*, 1993; Brändström *et al.*, 1994; Gleave *et al.*, 1992). It is therefore of interest to examine why apoptosis does not occur after castration and if it can be induced by additional treatments in an androgen-sensitive prostatic tumour. Interestingly, treatment with oestrogen has been shown to induce single-cell pyknosis in human prostatic tumours (Schenken *et al.*, 1943), to reduce the number of epithelial cells in the Dunning R3327PAP tumour (Landström *et al.*, 1990) and to increase the number of morphologically apoptotic cells in Dunning R3327PAP tumours relapsing after castration treatment (Landström *et al.*, 1994). The present study was therefore designed to compare short-term effects of oestrogen treatment in combination with castration with castration treatment alone in the Dunning R3327 PAP prostatic tumour model.

Correspondence: A Bergh
Received 2 September 1994; revised 10 February 1995; accepted 14 February 1995

### Materials and methods

### *Animals, treatments and tissue preparation*

Ten-week-old male Copenhagen/Fisher rats supplied from Møllegaard, Copenhagen, Denmark were inoculated bilaterally with small pieces of the highly differentiated, androgen-sensitive Dunning R3327PAP prostatic adenocarcinoma (originally obtained from Dr Norman N Altman, the Papanicolau Cancer Research Institute, Miami, FL, USA). The rats were kept in a controlled environment (12 h dark/12 h light) and fed rat pellets and tap water *ad libitum*. When the rats weighed about 400 g and the tumours had reached similar sizes (about 1500 $mm^3$), about 3 months after inoculation, they were randomly divided into three groups. The rats in two of these groups were castrated via the scrotal route. The others served as controls. Of the 40 castrated rats, 20 were treated with daily subcutaneous (s.c.) injections of 50 μg of oestradiol benzoate (Sigma, USA) in sesame oil (C + E2), and 20 with only sesame oil s.c.(C). Of the remaining 25 non-castrated rats, five were killed before therapy and the remainder were injected daily with sesame oil (I). After 12, 24, 72 or 168 h, five rats from each of the three groups were sacrificed by decapitation. The tumours were then removed and divided into small pieces. Randomly, from each tumour:

(1) Two pieces were put in Bouin's solution, dehydrated and embedded in methacrylate plastic, cut into 2-μm-thick sections and stained with eosin for light microscopic examination of volume densities of different tumour compartments (see below).
(2) Five small tissue pieces were fixed by immersion in 4% formaldehyde, 3% glutaraldehyde and 0.5% picric acid in cacodylate buffer, post fixed in 1% osmium tetroxide, dehydrated, embedded in Epon and cut into 1-μm-thick sections, which were stained with toluidine blue for light microscopical demonstration of mitotic and apoptotic cells (see below).

(3) One piece was put in methanol and one in buffered formalin for 24 h. These tissue pieces were then dehydrated, embedded in paraffin and cut into 4-μm-thick sections for immunohistochemical and *in situ* end-labelling (ISEL) examinations (see below).

*Tumour volume, morphology and morphometry*

Before castration and before killing the animals, tumour volumes (L × W × H × 0.5236) were measured with a micro-calliper (Landström *et al.*, 1990). This estimation of tumour volume is highly correlated with tumour weight (Landström *et al.*, 1990). The volume densities of epithelium, glandular lumina and stroma were determined using a point counting method; a square lattice was mounted in the eyepiece of a light microscope and hits falling over the different tissue compartments were counted as earlier described (Landström *et al.*, 1990).

Mitotic and apoptotic indices (percentage of apoptotic and mitotic cells) were determined by counting 3000 epithelial and 1500 stromal cells per tumour at 1000 × magnification. Apoptotic cells were defined as single rounded cells or fragments with densely aggregated chromatin and condensed cytoplasm, often lying in 'halos' of extracellular space (Kerr *et al.*, 1972). If more than one apoptotic body was seen per 'halo', these were considered to originate from the same cell and counted as one.

*Detection of proliferating cells*

For immunohistochemical detection of proliferating (cycling) cells, a monoclonal anti-proliferating cell nuclear antigen (PCNA) antibody was used (Landberg and Roos, 1991). Sections of the paraffin-embedded, methanol-fixed tumour pieces were deparaffinised, treated again with methanol to suppress endogenous peroxidase activity, washed with phosphate-buffered saline (PBS) and incubated overnight with a primary monoclonal anti-PCNA antibody (Dako M879, Dakopatts, Denmark). The sections were then incubated with a secondary biotinylated antibody for 30 min, followed by ABC reagents for 45 min and with peroxidase substrate for development for 15 min. Between incubations, the sections were washed for 10 min in PBS. After immunodetection, the sections were lightly counterstained with Meyer's haematoxylin solution. PCNA indices (percentage of PCNA labelled cells) were determined by counting 900 epithelial and 300 stromal cells per tumour at 1000 × magnification.

*In situ detection of apoptotic cells*

Along with the time-and labour-consuming electron microscopic detection of apoptotic cells, the novel and more convenient (ISEL or *in situ* nick translation methods are considered to be the most accurate in detecting apoptotic cells (Ansari *et al.*, 1993; Wijsman *et al.*, 1993). Basically, the protocol by Wijsman *et al.* was followed.

After dewaxing and rehydration of formalin-fixed tissue according to standard procedures, the sections were heated in 2 × SSC (0.3 M sodium chloride and 30 mM sodium citrate), pH 7, at 80°C for 20 min and subsequently washed thoroughly in distilled water. To enable enzymatic incorporation of nucleotides, the sections were digested in 0.5% pepsin in hydrochloric acid (pH 2) for 15 min with gentle shaking in a 37°C water bath. The digestion was stopped by washing several times in tap water and then in buffer A [50 mM Tris-HCl, 5 mM magnesium chloride, 10 mM β-mercaptoethanol and 0.005% bovine serum albumin (BSA; Sigma), pH 7.5] for 5 min. After drying, the sections were incubated for 1 h at 15°C with buffer A containing 0.01 mM dATP, dGTP, dCTP 0.01 mM biotin dUTP (Boehringer-Mannheim) and 4 U $ml^{-1}$ DNA polymerase I (Sigma). After blocking endogenous peroxidase for 5 min in 0.1% hydrogen peroxide in PBS, the sections were washed twice for 5 min in 0.1% hydrogen peroxide in PBS. The sections were then incubated with avidin (Boehringer-Mannheim) dissolved in PBS containing 1% BSA and 0.5% Tween 20 for 30 min at room temperature before developing with diaminobenzidine (Sigma). For negative controls, DNA polymerase was excluded from the nucleotide-polymerase mix. For positive controls, normal rat prostate was used 3 days after castration since about 4% of the epithelial cells are known to be apoptotic at this time (English *et al.*, 1989). ISEL indexes (percentage of ISEL-positive cells) were determined by counting approximately 3000 cells per tumour at 1000 × magnification.

*Statistics*

For comparisons between groups the Mann-Whitney *U*-test was employed. Correlation was expressed using linear regression analysis. A *P*-value of less than 0.05 was considered statistically significant.

### Results

*Tumour volume*

In the vehicle-treated intact animals the tumour volume increased during the study period (Figure 1). Castration inhibited tumour growth and the tumour volume remained constant. In contrast, castration plus oestrogen treatment reduced tumour volume from 72 h after treatment (Figure 1).

*Tumour morphology and composition*

The volume densities of epithelium, stroma and glandular lumina (not shown) remained constant during the study period in the intact and the castrated group (Figure 2a and b). In contrast, in the castrated + oestrogen-treated group the volume densities of tumour epithelium decreased and stroma increased as early as after 24 h of treatment (Figure 2a and b). The volume density of lumina was, however, unaffected (not shown). Necrotic tumour areas were rarely observed in intact, castrated, or castrated + oestrogen-treated tumours.

*Mitotic and PCNA labelling index*

The calculated PCNA labelling indices and mitotic indexes in the individual tumours were highly correlated ($r = 0.98$, $P < 0.00001$, $n = 65$). Therefore, only the data on

**Figure 1** Relative changes in average tumour volume of individual Dunning tumours treated with vehicle (I), castration + vehicle (C) and with castration + oestrogen (C + E2) after 0, 12, 24, 72 and 168 h of treatment. Each point in the diagram represents 7-10 tumours. a = significantly different from I group, b = significantly different from C group, $P < 0.05$ according to Mann-Whitney *U*-test. Error bars represent s.e.m. For details, see the Materials and methods and Results sections.

PCNA labelling are presented. The number of mitotic or PCNA-labelled stroma cells remained constant during the study period in the intact or in the castrated group (Figures 3a and 6). In the castrated + oestrogen-treated group these values increased considerably as early as 12 h after treatment, but they returned to basal values at 168 h (Figures 3a and 6). The number of mitotic or PCNA-labelled tumour epithelial cells did not change in intact animals, but in castrated animals it decreased at 72 and 168 h (Figure 3b). In the castrated + oestrogen-treated animals there was an even more marked reduction in epithelial cell proliferation at 72 and 168 h (Figure 3b). In all groups the percentage of PCNA-labelled tumour epithelial cells correlated with the relative change in tumour volume during the study period ($r = 0.51$, $P < 0.0001$). It should be noted that PCNA index may increase under some circumstances in non-proliferating cells (Harrison *et al*., 1993). However, the correlation between PCNA and mitotic indexes suggest that the PCNA-labelling index is related to cell proliferation in the circumstances studied here.

*Apoptosis*

Apoptotic/ISEL-positive cells were extremely rare outside the epithelial compartment. The epithelial cell apoptotic and ISEL indexes in individual tumours were highly correlated ($r = 0.99$, $P < 0.0001$, $n = 30$). For this reason we only performed ISEL in the 12 and 24 h groups. The ISEL and apoptotic indexes were unchanged during the study period in the intact group (Figures 4 and 5). Castration resulted in a significant reduction in the number of apoptotic cells (compared with the I and C + E2 groups) starting as early as 12 h after treatment. The apoptotic indexes (ISEL indexes at 24 h) were significantly higher in the C + E2 than in the C and I groups at 24, 72 and 168 h after treatment (Figures 4 and 6). There was a large increase in the number of ISEL-positive cells in the ventral prostate 3 days after castration (data not shown), confirming the effectiveness of the ISEL method in detecting apoptotic cells. The rarely observed necrotic areas

**Figure 2** Stromal (**a**) and epithelial (**b**) volume density of individual Dunning tumours treated with vehicle (I), castration + vehicle (C) and with castration + oestrogen (C + E2) after 0, 12, 24, 72 and 168 h of treatment. Each point in the diagram represents 4–6 tumours. a = significantly different from I group, b = significantly different from C group, $P < 0.05$ according to Mann–Whitney *U*-test. Error bars represent s.e.m. For details, see the Materials and methods and Results sections.

**Figure 3** Stromal (**a**) and epithelial (**b**) PCNA incorporation (**a**) in individual Dunning tumours treated with vehicle (I), castration + vehicle (C) and with castration + oestrogen (C + E2) after 0, 12, 24, 72 and 168 h of treatment. Each point in the diagram represents 4–6 tumours. a = significantly different from I group, b = significantly different from C group, $P < 0.05$ according to Mann–Whitney *U*-test. Error bars represent s.e.m. For details, see the Materials and methods and Results sections.

**Figure 4** Tumour epithelial cell apoptotic indexes of individual Dunning tumours treated with vehicle (I), castration + vehicle (C) and with castration + oestrogen (C + E2) after 0, 12, 24, 72 and 168 h of treatment. Each point in the diagram represents 4–8 tumours. a = significantly different from I group, b = significantly different from C-group, $P < 0.05$ according to Mann–Whitney *U*-test. Error bars represent s.e.m. For details, see the Materials and methods and Results sections.

**Figure 5** *In situ* end labelling (ISEL) indexes of individual Dunning tumours treated with vehicle (I), castration + vehicle (C) and with castration + oestrogen (C + E2) after 12 and 24 h of treatment. Each bar in the diagram represents 5–7 tumours. a = significantly different from I group, b = significantly different from C group, $P < 0.05$ according to Mann–Whitney *U*-test. Error bars represent s.e.m. For details, see the Materials and methods and Results sections.

were also stained by ISEL but were easily distinguished from apoptotic cells.

### Discussion

One important observation in this study is that castration reduces the apoptotic index and therefore probably the number of apoptotic epithelial cells in the androgen-sensitive Dunning R3327PAP tumour. We have previously suggested that the principal reason for the inhibited tumour growth observed in this model after castration could be a decreased epithelial cell proliferation rate and epithelial cell atrophy, and not an increased apoptotic rate (Westin *et al.*, 1993; Brändström *et al.*, 1994). The present findings support this conclusion and offer an explanation for our previous finding that, in spite of a reduced cell proliferation rate, epithelial cell numbers are unaffected compared with that in untreated tumours up to 6 weeks after castration in this tumour model (Landström *et al.*, 1990). Moreover, as aberrant cell survival can contribute to tumour progression in different ways (Carson and Ribeiro, 1993), it is not surprising that the inhibitory a b c d e f

**Figure 6** Section from Dunning tumours treated for 24 h with castration + vehicle (**a**, **c**, and **e**) and castration + oestrogen (**b**, **d**, and **f**). (**a** and **b**) Epon sections, 700 × magnification. Arrowheads on apoptotic cells. Considerably more apoptotic cells are found in C + E2-treated tumours (**b**), which also show proliferating stromal cells (arrow on mitotic large stromal cell). Apoptotic cells are found in the epithelium. (**c** and **d**) Sections from *in situ* end labelling of apoptotic cells, 700 × magnification. Arrowheads on stained apoptotic cells. Considerably more stained cells are found in C + E2-treated tumours (**d**). Staining cells are of epithelial origin. (**e** and **f**) Sections from immunohistochemical labelling of cycling cells with monoclonal antibodies to PCNA, 175 × magnification. Arrows on stained stromal cells (large stromal cells and fibroblasts). Considerably more stromal cells are stained in C + E2-treated tumours (**f**).

effect of castration on tumour growth rate is transient in this tumour model (Landström *et al.*, 1994). It has previously been shown that androgens stimulate cell proliferation and inhibit cell death in the rat ventral prostate (Isaacs, 1984). The present observation suggests that cell proliferation, but not cell death, may be controlled in the same way in the Dunning R3327PAP tumour model. The reason why regulation of cell death and proliferation are apparently dissociated in this tumour remains to be studied.

According to current opinion, prostatic tumours that respond to androgen ablation therapy are composed of androgen-dependent and androgen-independent cells. The androgen-dependent cells presumably die after androgen ablation therapy and the independent ones survive and eventually repopulate the tumour (Isaacs and Coffey, 1981). However, this concept may not be valid for the Dunning R3327PAP tumour. There are no indications that androgen-dependent cells are present in this tumour as there are no signs of castration-induced cell death. The tumour cells are not androgen independent since they do respond to treatment with a decreased proliferation rate and atrophy (Landström *et al.*, 1990; Westin *et al.*, 1993). It therefore appears that this tumour is composed mainly of an androgen-sensitive cell population. How relevant then is this tumour model for human prostatic cancer? This question cannot be answered at present. Preliminary data do, however, indicate that castration therapy causes tumour cell atrophy and decreased cell proliferation rate, but not apoptotic cell death, in several prostatic cancer patients and that a subgroup of these tumours may respond with a decreased apoptotic rate (Westin *et al.*, 1995).

Another interesting finding in the present study is that combined oestrogen and castration treatments result in a 3-fold increase in cell death rate 24 h after the start of treatment, which is then maintained at a significantly higher level than in untreated tumours. Theoretically, an increased apoptotic index could reflect a decreased rate at which apoptotic bodies are phagocytosed. However, as tumour volume and epithelial volume density were decreased, a more likely explanation for the observed increase in the number of apoptotic epithelial cells in these tumours is that more cells are actually dying by apoptosis. The magnitude of the apoptotic response to oestrogen treatment, though relatively small, is significant since it, together with the decrease in cell proliferation and possibly in cell size (Landström *et al.*, 1990), results in a 30% decrease in tumour volume during the study period.

The observation that oestrogen + castration treatment may induce apoptosis in an androgen-sensitive prostatic cancer model is interesting as it suggests that there could be additional perhaps more effective ways than castration to induce cell death in prostatic tumours.

Oestrogen may act in several ways. It may have direct effects on the tumour epithelial cells, as suggested by the observation of apoptosis in a prostatic tumour cell line after treatment with diethylstilboestrol *in vitro* (Fine *et al.*, 1994). The mechanism behind this is unknown, but oestrogen treatment is able to release intracellular calcium levels via an unconventional cell-surface oestrogen receptor in granulosa cells (Morley *et al.*, 1992), and increases in intracellular $Ca^{2+}$ may activate key enzymes such as endonuclease and tissue transglutaminase involved in apoptosis (Fesus *et al.*, 1991). However, observations in this study suggest that the effect of oestrogen could be secondary to effects in the tumour stroma; the increase in epithelial cell apoptosis was preceded by a proliferative response in the stroma. Moreover, oestrogen receptors in the Dunning R3327PAP tumour are located mainly in the stroma (Markland *et al.*, 1978; Beckman *et al.*, 1985), and it has also been shown that oestrogen exerts stimulatory effects on the stroma of the normal prostate (Krieg *et al.*, 1983) as well as that in Dunning R3327PAP tumours (Landström *et al.*, 1990). Interestingly, it has also been noted that signs of increased activity in stromal cells occur simultaneously with epithelial cell apoptosis after castration in the normal prostate (Bacher *et al.*, 1993; Zhao *et al.*, 1993) and in the PC-82 human prostatic tumour model (vanWerden *et al.*, 1993). Stromal cells in all tissues may, by providing 'survival signals', regulate cell death rate in the adjacent parenchymal cells (Raff, 1992). By secreting stimulatory and inhibitory factors and by providing extracellular matrix stroma cells may regulate epithelial cell survival, differentiation and growth in the normal, hyperplastic and malignant prostate (Oosterwijk-König *et al.*, 1985; Cunha *et al.*, 1986; König *et al.*, 1987; Chung, 1993; Meredith *et al.*, 1993; Frisch and Francis, 1994). We therefore suggest that the reason why combined oestrogen and castration treatment, in contrast to castration alone, induces epithelial cell apoptosis in the Dunning R3327PAP tumour is that it has a stimulatory effect on its stroma. This hypothesis is currently under investigation.

**Acknowledgements**

Mrs Birgitta Ekblom, Mrs Sigrid Kilter and Mrs Elisabeth Dahlberg have contributed to this paper by their skilful technical assistance. This study was supported by grants from the Swedish Cancer Society (Project No. 1760), the Maud and Birger Gustavsson Foundation, the Sahlberg Foundation and the Lion's Research Foundation, Umeå.

### References

ANSARI B, COATES PJ, GREENSTEIN BD AND HALL PA. (1993). *In situ* end labelling detects DNA strand breaks in apoptosis and other physiological and pathological states. *J. Pathol.*, **170**, 1-8.

BACHER, RAUSCH UM, GOEBEL H-W, POLZAR B, MANNHERZ HG AND AUMÜLLER G. (1993). II. Stromal and epithelial cells from rat prostate during androgen deprivation and estrogen treatment – regulation of transcription. *Exp. Clin. Endocrinol.*, **101**, 78-86.

BECKMAN JR WC, MICKEY DD AND FRIED FA. (1985). Autoradiographic localisation of estrogen and androgen target cells in human and rat prostatic carcinoma. *J. Urol.*, **133**, 724-728.

BRÄNDSTRÖM A, WESTIN P, BERGH A, CAJANDER S AND DAMBER J-E. (1994). Castration induces apoptosis in the ventral prostate but not in an androgen-sensitive prostatic adenocarcinoma in the rat. *Cancer Res.*, **54**, 3594-3601.

CARSON DA AND RIBEIRO J. (1993). Apoptosis and disease. *Lancet*, **341**, 1251-1254.

CHUNG LWK. (1993). Implications of stromal-epithelial interaction in human prostate cancer growth. *Semin. Cancer Biol.*, **4**, 183-192.

CUNHA GR, DONJACOUR AA AND SUGIMURA Y. (1986). Stromal-epithelial interactions and heterogeneity of proliferative activity within the prostate. *Biochem. Cell Biol.*, **64**, 608-614.

ENGLISH HF, KYPRIANOU N AND ISAACS JT. (1989). Relationship between DNA fragmentation and apoptosis in the programmed cell death in the rat prostate following castration. *Prostate*, **15**, 233-250.

FESUS L, DAVIES PJA AND PIACENTINI M. (1991). Apoptosis: molecular mechanisms in progammed cell death. *Eur. J. Cell Biol.*, **56**, 170-177.

FINE RL, ROBERTSON KM, O'BRIEN T, PADILLA G AND ROBERTSON CN. (1994). High dose diethylstilbestrol (DES) induces cytotoxicity of human tumour cell lines in vitro via a mechanism involving apoptosis (abstract 1886). *Proc. Am. Assoc. Cancer Res.*, **35**.

FRISCH SM AND FRANCIS H. (1994). Disruption of epithelial cell-matrix interactions induces apoptosis. *J. Cell Biol.*, **124**, 619-626.

GLEAVE ME, HSIEH J-E, WU HC, VON ESCHENBACH AC AND CHUNG LWK. (1992). Serum prostate specific antigen in mice bearing human prostate LNCaP tumours determined by tumor volume and endocrine growth factors. *Cancer Res.*, **52**, 1598-1605.

HARRISON RF, REYNOLDS GM AND ROWLANDS DC. (1993). Immunohistochemical evidence for the expression of proliferating cell nuclear antigen (PCNA) by non-proliferating hepatocytes adjacent to metastatic tumours and inflammatory conditions. *J. Pathol.*, **171**, 115–122.

ISAACS JT. (1984). Antagonistic effects of androgen on prostatic cell death. *Prostate*, **5**, 545–557.

ISAACS JT AND COFFEY DS. (1981). Adaptation versus selection as the mechanism responsible for the relapse of prostatic cancer to androgen ablation therapy as studied in the Dunning R-3327H adenocarcinoma. *Cancer Res.*, **41**, 5070–5075.

KERR JF, WYLLIE AH AND CURRIE AR. (1972). Apoptosis: a basic biological phenomenon with wide-ranging implications in tissue kinetics. *Br. J. Cancer*, **26**, 239–257.

KRIEG M, BARTSCH W, THOSEN M AND VOIGHT KD. (1983). Androgens and estrogens: their interaction with stroma and epithelium of human benign prostatic hyperplasia and normal prostate. *J. Steroid Biochem.*, **19**, 155–161.

KYPRIANOU N AND ISAACS JT. (1988). Activation of programmed cell death in the rat ventral prostate after castration. *Endocrinology*, **122**, 552–562.

KYPRIANOU N, ENGLISH HF AND ISAACS JT. (1990). Programmed cell death during regression of PC-82 human prostate cancer following androgen ablation. *Cancer Res.*, **50**, 3748–3753.

KÖNIG JJ, ROMJIN JC AND SCHRÖDER FH. (1987). Prostatic epithelium inhibiting factor (PEIF): organ specificity and production by prostatic fibroblasts. *Urol. Res.*, **15**, 145–149.

LANDBERG G AND ROOS G. (1991). Antibodies to proliferating cell nuclear antigen (PCNA) as S-phase probes in flow cytometrical cell cycle analysis *Cancer Res.*, **51**, 4570–4574.

LANDSTRÖM M, BERGH A, TOMIC R AND DAMBER J-E. (1990). Estrogen treatment combined with castration inhibits tumor growth more effectively than castration alone in the Dunning R-3327 rat prostatic adenocarcinoma. *Prostate*, **17**, 57–68.

LANDSTRÖM M, DAMBER J-E AND BERGH A. (1994). Estrogen treatment postpones the castration induced dedifferentiation of Dunning R-3327PAP prostatic adenocarcinoma. *Prostate*, **25**, 10–18.

McDONNEL TJ AND KORSMEYER SJ. Progression from lymphoid hyperplasia to high grade malignant lymphoma in mice transgenic for the t(14:18). *Nature*, **349**, 254–256.

MARKLAND FJ, CHOPP R, COXGROVE MD AND HOWARD EB. (1978). Characterisation of steroid hormone receptors in the Dunning R-3327 rat prostatic carcinoma. *Cancer Res.*, **38**, 2818–2826.

MEREDITH JE JR, FAZELI B AND SCHWARTZ MA. (1993). The extracellular matrix as a cell survival factor. *Mol. Biol. Cell*, **4**, 953–961.

MORLEY P, WHITFIELD JF, VANDERHYDEN BC, TSANG BK AND SCHWARTZ J-L. A. (1992). New, non genomic estrogen action: the rapid release of intracellular calcium. *Endocrinology*, **131**, 1305–1312.

OOSTERWIJK-KÖNIG JJ, ROMJIN JC AND SCHRÖDER FH. (1985). Modulation by prostatic fibroblasts of the clonal growth of a prostatic carcinoma cell line (PC-3) in vitro. *Prostate*, **6**, 459–460.

RAFF MC. (1992). Social controls on cell survival and cell death. *Nature*, **356**, 397–399.

RAGHAVAN D. (1988). Non-hormone chemotherapy for prostate cancer: principles of treatment and application to the testing of new drugs. *Semin. Oncol.*, **15**, 371–389.

SCHENKEN JR, BURNS EL AND KAHLE PJ. (1943). The effect of diethylstilbestrol diproprionate on carcinoma of the prostatic gland. II. Cytological changes following treatment. *J. Urol.*, **48**, 99–112.

UMANSKY SR. (1982). The genetic program of cell death. Hypothesis and some applications: transformation, carcinogenesis, ageing. *J. Theor. Biol.*, **97**, 591–602.

VANWERDEN WM, VAN KREUNINGEN A, ELISSEN NMJ, VERMEIJ M, DE JONG FH, VAN STEENBRUGGE GJ AND SCHRÖDER FH. (1993). Castration-induced changes in morphology, androgen levels, and proliferative activity of human prostate cancer tissue grown in athymic nude mice. *Prostate*, **23**, 149–164.

WESTIN P, BERGH A AND DAMBER J-E. (1993). Castration rapidly results in a major reduction in epithelial cell numbers in the rat prostate but not in the highly differentiated Dunning R-3327PAP prostatic adenocarcinoma. *Prostate*, **22**, 65–74.

WESTIN P, STATTIN P, DAMBER J-E AND BERGH A. (1995). Castration therapy rapidly induces apoptosis in a minority and decreases cell proliferation in a majority of human prostatic tumours. *Am. J. Pathol.* (in press).

WIJSMAN JH, JONKER RR, KEIJZER R, VAN DE VELDE CJH, CORNELISSE CJ AND DIERENDONCK JH. (1993). A new method to detect apoptosis in paraffin sections: *in situ* end-labelling of fragmented DNA. *J. Histochem. Cytochem.*, **41**, 7–12.

ZHAO GQ, BACHER M, FRIEDRICHS W, SCHMIDT W, RAUSCH U, GOEBEL H-W, TUOHIMAA P AND AÜMULLER G. (1993). I. Functional properties of isolated stroma and epithelium from rat ventral prostate during androgen deprivation and estrogen treatment. *Exp. Clin. Endocrinol.*, **101**, 69–77.